# Supplementary material for: Non-Synonymous Variants in Fat QTL Genes among High- and Low-Milk-Yielding Indigenous Breeds
Source: Animals (Basel). 2023 Feb 28;13(5):884. doi: 10.3390/ani13050884 (PMC10000039; doi:10.3390/ani13050884)
Supplement: Supplementary file 1 [file animals-13-00884-s001.zip › Supplementary_files/SM-5.docx]

**Table S1.** List of metabolic genes associated with milk fat traits.

| Gene Ids | Gene Name | Gene Description |
| --- | --- | --- |
| ENSBTAG00000011885 | *NNT* | Nicotinamide nucleotide transhydrogenase [Source:VGNC Symbol;Acc:VGNC:32143] |
| ENSBTAG00000006429 | *ACO2* | Aconitase 2 [Source:VGNC Symbol;Acc:VGNC:25546] |
| ENSBTAG00000015980 | *FASN* | Fatty acid synthase [Source:VGNC Symbol;Acc:VGNC:28871] |
| ENSBTAG00000020527 | *IDH1* | isocitrate dehydrogenase (NADP (+)) 1 [Source:NCBI gene;Acc:281235] |
| ENSBTAG00000048655 | *NT5E* | 5'-nucleotidase ecto [Source:NCBI gene;Acc:281363] |
| ENSBTAG00000014521 | *UGDH* | UDP-glucose 6-dehydrogenase [Source:VGNC Symbol;Acc:VGNC:36648] |
| ENSBTAG00000047039 | *CYP21* | Bos taurus cytochrome P450, family 21, subfamily A, polypeptide 2 (CYP21A2), mRNA. [Source:RefSeq mRNA;Acc:NM_174639] |
| ENSBTAG00000010449 | *MOCS1* | Molybdenum cofactor synthesis 1 [Source:VGNC Symbol;Acc:VGNC:54229] |
| ENSBTAG00000001156 | *ST3GAL1* | ST3 beta-galactoside alpha-2,3-sialyltransferase 1 [Source:VGNC Symbol;Acc:VGNC:35331] |
| ENSBTAG00000000593 | *ST8SIA1* | ST8 alpha-N-acetyl-neuraminide alpha-2,8-sialyltransferase 1 [Source:VGNC Symbol;Acc:VGNC:49964] |
| ENSBTAG00000026356 | *DGAT1* | Diacylglycerol O-acyltransferase 1 [Source:VGNC Symbol;Acc:VGNC:28020] |
| ENSBTAG00000026181 | *UGT1A1* | UDP glucuronosyltransferase 1 family, polypeptide A6 [Source:NCBI gene;Acc:286792] |
| ENSBTAG00000008338 | *PLCB1* | Phospholipase C beta 1 [Source:VGNC Symbol;Acc:VGNC:32980] |
| ENSBTAG00000005735 | *ATP5MC2* | ATP synthase membrane subunit c locus 2 [Source:VGNC Symbol;Acc:VGNC:55848] |
| ENSBTAG00000017281 | *OPLAH* | 5-oxoprolinase, ATP-hydrolysing [Source:VGNC Symbol;Acc:VGNC:53869] |
| ENSBTAG00000006579 | *P4HA3* | prolyl 4-hydroxylase subunit alpha 3 [Source:VGNC Symbol;Acc:VGNC:32537] |
| ENSBTAG00000013825 | *BCAT1* | branched chain amino acid transaminase 1 [Source:VGNC Symbol;Acc:VGNC:26439] |
| ENSBTAG00000012794 | *PAH* | Phenylalanine hydroxylase [Source:VGNC Symbol;Acc:VGNC:32554] |
| ENSBTAG00000023939 | ENSBTAG00000023939 | Cytochrome P450, family 2, subfamily c [Source:NCBI gene;Acc:511498] |
| ENSBTAG00000005730 | *GPAT4* | Glycerol-3-phosphate acyltransferase 4 [Source:NCBI gene;Acc:511614] |
| ENSBTAG00000004040 | *UGT2A1* | UDP glucuronosyltransferase family 2 member A1 complex locus [Source:NCBI gene;Acc:511743] |
| ENSBTAG00000013489 | *CYP27A1* | cytochrome P450, family 27, subfamily A, polypeptide 1 [Source:NCBI gene;Acc:511960] |
| ENSBTAG00000002827 | *ACAT2* | acetyl-CoA acetyltransferase 2 [Source:VGNC Symbol;Acc:VGNC:25530] |
| ENSBTAG00000020715 | *PIK3C2G* | phosphatidylinositol-4-phosphate 3-kinase catalytic subunit type 2 gamma [Source:VGNC Symbol;Acc:VGNC:32887] |
| ENSBTAG00000022058 | *ACACB* | acetyl-CoA carboxylase beta [Source:VGNC Symbol;Acc:VGNC:25520] |
| ENSBTAG00000007829 | *CSAD* | cysteine sulfinic acid decarboxylase [Source:VGNC Symbol;Acc:VGNC:27749] |
| ENSBTAG00000045703 | *COX15* | cytochrome c oxidase assembly homolog COX15 [Source:VGNC Symbol;Acc:VGNC:27629] |
| ENSBTAG00000005091 | *DGKG* | diacylglycerol kinase gamma [Source:VGNC Symbol;Acc:VGNC:28028] |
| ENSBTAG00000018966 | *PLCE1* | phospholipase C epsilon 1 [Source:VGNC Symbol;Acc:VGNC:32987] |
| ENSBTAG00000016663 | *PIGN* | phosphatidylinositol glycan anchor biosynthesis class N [Source:VGNC Symbol;Acc:VGNC:32873] |
| ENSBTAG00000008553 | *B4GALNT3* | beta-1,4-N-acetyl-galactosaminyltransferase 3 [Source:VGNC Symbol;Acc:VGNC:26387] |
| ENSBTAG00000010564 | *ELOVL6* | ELOVL fatty acid elongase 6 [Source:VGNC Symbol;Acc:VGNC:28452] |
| ENSBTAG00000001030 | *MTMR3* | Myotubularin related protein 3 [Source:VGNC Symbol;Acc:VGNC:31742] |
| ENSBTAG00000014841 | *GBA* | Glucosylceramidase beta [Source:VGNC Symbol;Acc:VGNC:50183] |
| ENSBTAG00000007689 | *LPIN1* | Lipin 1 [Source:VGNC Symbol;Acc:VGNC:30966] |
| ENSBTAG00000016867 | *HSD17B3* | Hydroxysteroid 17-beta dehydrogenase 3 [Source:VGNC Symbol;Acc:VGNC:29974] |
| ENSBTAG00000005989 | *LAP3* | Leucine aminopeptidase 3 [Source:VGNC Symbol;Acc:VGNC:30786] |
| ENSBTAG00000049496 | ENSBTAG00000049496 | Cytochrome P450 2C31 [Source:NCBI gene;Acc:785540] |

**Table S2.** List of top twenty most expressed genes in Jersey and Kashmiri breeds with respective average expression for breeds

| **Jersey** | | **Kashmiri** | |
| --- | --- | --- | --- |
| **Gene Name** | **Expression** | **Gene Name** | **Expression** |
| Beta-lactoglobulin (LOC113901792) | 782314.80 | Beta-lactoglobulin (LOC113901792) | 2685945.08 |
| Casein beta (CSN2) | 361401.56 | Casein beta (CSN2) | 1090101.13 |
| Casein alpha s1 (CSN1S1) | 306134.56 | Casein alpha s1 (CSN1S1) | 1030232.03 |
| Interleukin 1 receptor antagonist (IL1RN) | 257117.12 | Lactalbumin alpha (LALBA) | 434015.42 |
| Serine dehydratase (SDS) | 239347.35 | Casein kappa (CSN3) | 258469.31 |
| Transglutaminase 3 (TGM3) | 180128.97 | Eukaryotic translation elongation factor 1 alpha 1 (EEF1A1) | 201756.92 |
| Ferritin heavy chain 1 (FTH1) | 142092.04 | Glycosylation-dependent cell adhesion molecule 1 (LOC113893789) | 142399.03 |
| Solute carrier family 2 member 3 (SLC2A3) | 130612.05 | Alpha-S2-casein (LOC113894643) | 113610.35 |
| Casein kappa (CSN3) | 114734.89 | Tumor protein, translationally controlled 1 (TPT1) | 110854.31 |
| C-X-C motif chemokine receptor 2 (CXCR2) | 111639.96 | Ribosomal protein lateral stalk subunit P0 (RPLP0) | 107345.84 |
| Serglycin (SRGN) | 107447.66 | Ribosomal protein S2 (RPS2) | 102623.21 |
| Cathepsin B (CTSB) | 97513.33 | Ribosomal protein lateral stalk subunit P1 (RPLP1) | 86088.89 |
| BOLA class I histocompatibility antigen (LOC113882062) | 97190.73 | Collagen alpha-1(I) chain-like (LOC113889184) | 82942.67 |
| C-X-C motif chemokine ligand 8 (CXCL8) | 91813.35 | Ribosomal protein (RPL10) | 74930.51 |
| Secreted phosphoprotein 1 (SPP1) | 90319.64 | Ribosomal protein SA (RPSA) | 71474.73 |
| Lactalbumin alpha (LALBA) | 84506.65 | Ribosomal protein L13A (RPL13A) | 68664.24 |
| LOC113899279 | 82523.72 | Ribosomal protein S3A (RPS3A) | 66853.11 |
| LOC113893789 | 80362.57 | Fatty acid synthase (FASN) | 65660.55 |
| Eukaryotic translation factor 1 alpha 1 (EEF1A1) | 79147.98 | Ribosomal protein L13 (RPL13) | 63426.11 |
| LOC113884060 | 78473.06 | Secreted phosphoprotein 1SPP1 | 62896.44 |

**Table S3.** List of top ten up-and down regulated differentially expressed genes between Jersey and Kashmiri breed along with QTL id and trait name.

| **S.N.** | **Gene** | **Padj-value** | **Log2FC** | **QTL id** | **Trait name** | **Description** |
| --- | --- | --- | --- | --- | --- | --- |
| **Upregulated genes** | | | | | | |
| 1 | *CXCL8* | 7.70 | 7.10 | 9939 | MFY | C-X-C motif chemokine ligand 8 |
| 2 | *ETS2* | 6.80 | 4.77 | 136176 | MFP | ETS proto-oncogene 2, transcription factor |
| 3 | *SLC6A9* | 1.01 | 4.50 | 47483 | MFY | Solute carrier family 6-member 9 |
| 4 | *KCNK9* | 3.83 | 4.43 | 33380,  174222 | MFP, MFY | Potassium two pore domain channel subfamily K member 9 |
| 5 | *TG* | 1.27 | 4.36 | 173769 | MFP | Thyroglobulin |
| 6 | *DGKG* | 4.42 | 4.03 | 136180 | MFP | Diacylglycerol kinase gamma |
| 7 | *TLR4* | 1.23 | 3.92 | 14033 | MFP | Toll like receptor 4 |
| 8 | *SLC45A4* | 5.08 | 3.69 | 173648,  174201 | MFP, MFY | Solute carrier family 45-member 4 |
| 9 | *OLR1* | 6.92 | 3.69 | 136185 | MFP | Oxidized low density lipoprotein receptor 1 |
| 10 | *PARM1* | 2.98 | 3.64 | 57254 | MFY | Prostate androgen-regulated mucin-like protein 1 |
| **Downregulated genes** | | | | | | |
| 1 | *MTMR12* | 4.54 | -4.30 | 173951 | MFP | Myotubularin related protein 12 |
| 2 | *GRHL3* | 1.23 | -2.97 | 173446 | MFP | Grainyhead like transcription factor 3 |
| 3 | *ARHGAP39* | 6.63 | -2.90 | 161665 | MFP | Rho GTPase activating protein 39 |
| 4 | *CCND3* | 1.35 | -2.89 | 175837 | MFP | Cyclin D3 |
| 5 | *MATN2* | 5.49 | -2.82 | 173803 | MFP | Matrilin 2 |
| 6 | *RAI14* | 9.78 | -2.75 | 173946 | MFP | Retinoic acid induced 14 |
| 7 | *ELOVL6* | 3.22 | -2.59 | 161548 | MFP | ELOVL fatty acid elongase 6 |
| 8 | *CD2* | 2.15 | -2.55 | 57255 | MFP | CD2 molecule |
| 9 | *MFGE8* | 1.57 | -2.54 | 51082 | MFP | Milk fat globule EGF and factor V/VIII domain containing |
| 10 | *P4HA3* | 4.23 | -2.44 | 173814 | MFP | Prolyl 4-hydroxylase subunit alpha 3 |

MFP: milk fat percentage, MFY:Milk fat yield, Log2FC: log2 fold change, Padj-value: p-value adjusted

**Figure S1.** The schematic representation of the whole study.

 **Figure S2**. Quality evaluation of samples. (**A**) Library size of sequenced samples. (**B**) Boxplot of logCPM expression values across the samples. (**C**) PCA and (**D**) MDS plot of normalized expression values of samples. Jersey, and Kashmiri were represented through green and red colours respectively.
